# Acalculous Cholecystitis: An Uncommon Presentation of Babesiosis

**DOI:** 10.7759/cureus.22165

**Published:** 2022-02-13

**Authors:** Ayham Khrais, Dhanasekaran Ramasamy, Shiva Kumar

**Affiliations:** 1 Internal Medicine, Rutgers University, New Jersey Medical School, Newark, USA; 2 Department of Gastroenterology, Cooperman Barnabas Medical Center, Center for Digestive Diseases, Union, USA; 3 Department of Gastroenterology and Hepatology, Cleveland Clinic Abu Dhabi, Digestive Diseases Institute, Abu Dhabi, ARE

**Keywords:** gallbladder disorders, hemolysis, acute acalculous cholecystitis, tick borne infections, babesiosis

## Abstract

Human babesiosis is commonly caused by *Babesia microti*, an infectious protozoan with a preference for erythrocytes. We describe a case of babesiosis presenting with acute acalculous cholecystitis. A 74-year-old man with a history of diabetes mellitus presented with four days of fever, chills, dyspnea on exertion, and dark brown urine. A physical exam was notable for scleral icterus. Laboratory findings were significant for lactate dehydrogenase (LDH) of 518, total bilirubin of 7.4, and direct bilirubin of 6.2. Imaging, including abdominal ultrasound, CT abdomen and pelvis, magnetic resonance cholangiopancreatography (MRCP), and hepatobiliary iminodiacetic acid (HIDA) scans, demonstrated acute acalculous cholecystitis. On further history, the patient confirmed a recent hiking trip in Virginia. Further evaluation, including peripheral smear and polymerase chain reaction (PCR), was consistent with *Babesia microti* infection. Babesiosis is common in the Northeastern and Midwestern United States, and symptoms can range from asymptomatic infection to nonspecific malaise and fever to severe end-organ dysfunction. Diagnosis is via peripheral smear or PCR, which can be confirmed via serology. The combination of clindamycin and quinine or atovaquone and azithromycin are the cornerstones of pharmacotherapy. Acute acalculous cholecystitis is a very uncommon presentation of babesiosis. Babesia infection must be considered in the differential in a patient with nonspecific symptoms living in an endemic area.

## Introduction

Babesiosis is a disease caused by organisms of the *Babesia* genus, which are intracellular protozoa with a tropism for red blood cells [1]. Of the various species of *Babesia*, *B. microti* is the most common disease-causing organism in humans [1,2]. Approximately 1,000 to 2,000 individuals are diagnosed with babesiosis in the United States annually, with disease incidence rising steadily [1]. The protozoa are transmitted primarily by *Ixodes scapularis *[1-6]. Clinical features range from no symptoms to nonspecific symptoms, including fatigue, malaise, weakness, and fever, to severe symptoms such as acute respiratory distress syndrome (ARDS), renal failure, hemolytic anemia with thrombocytopenia, and acute liver failure [1,4]. Hemolytic anemia may result in elevated bilirubin levels, which can be mistaken for a biliary tract or liver pathology. Diagnosis is via peripheral blood smear demonstrating intraerythrocytic organisms, polymerase chain reaction (PCR) testing, or a four-fold increase in antibodies directed against the protozoan. Treatment may involve a combination of clindamycin and quinine or atovaquone and azithromycin [3,4]. We describe a case of babesiosis presenting with acute acalculous cholecystitis. 

## Case presentation

A 74-year-old man with a history of diabetes mellitus presented with four days of fever, chills, dyspnea on exertion, and dark brown urine. He had tested negative for COVID-19 earlier. Physical examination revealed scleral icterus. Laboratory findings (Table 1) were significant for white blood cell count of 4,900 K/CMM, hemoglobin of 12.9 g/dL, serum aspartate aminotransferase (AST) of 79 Units/L, alanine aminotransferase (ALT) of 60 Units/L, lactate dehydrogenase (LDH) of 518 Units/L, D-dimer of 1,818 ng/mL, total bilirubin of 7.4 mg/dL and direct bilirubin of 6.2 mg/dL. Further evaluation included abdominal ultrasound (US), hepatobiliary iminodiacetic acid (HIDA) scan, magnetic resonance cholangiopancreatography (MRCP), and CT of abdomen/pelvis (CT A/P).

**Table 1:** Significant laboratory values, including results from CBC, CMP and parasitology CBC - complete blood count; CMP - comprehensive metabolic panel; RBC - red blood cells; WBC - white blood cells; MCV - mean corpuscular volume; PCR - polymerase chain reaction; IgM - immunoglobulin M; WB - western blot

	Value	Reference range
Hemoglobin	12.9 g/dL	13.5-17.0 g/dL
Hematocrit	37.7%	39.8-52%
RBC	4.45 MIL	4.5-5.9 MIL
WBC	4.9 K/CMM	4-10.5 K/CMM
Platelet	158 K/CMM	140-450 K/CMM
MCV	84.7%	81-100%
D-dimer	1,818 DDu ng/mL	≤ 243 DDu ng/mL
Aspartate aminotransferase	79 Unit/L	≤ 41 Unit/L
Alanine aminotransferase	60 Unit/L	≤ 45 Unit/L
Total bilirubin	7.4 mg/dL	0.0-1.0 mg/dL
Direct bilirubin	6.2 mg/dL	0.0-0.4 mg/dL
Lactate dehydrogenase	518 U/L	140-280 U/L
% Babesia parasitemia	1.0%	<0.0%
Babesia microti DNA	Detected	Not detected
Lyme PCR	Not detected	Not detected
Lyme IgM WB	Positive	Negative

**Figure 1:**
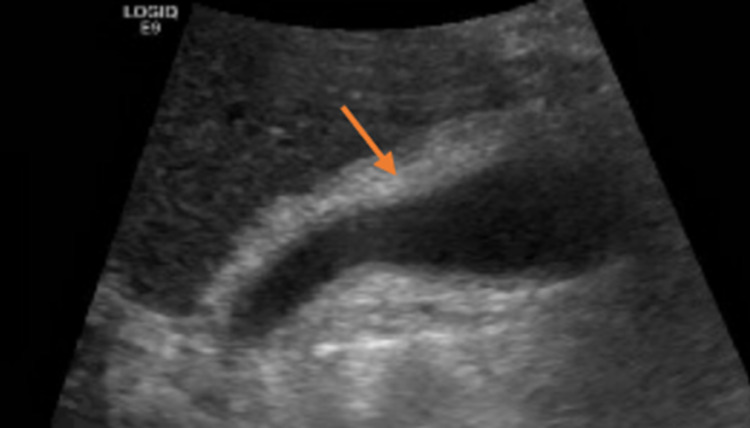
RUQ US demonstrating mild GB wall thickening (arrow) without evidence of gallstones RUQ - right upper quadrant; US - ultrasound; GB - gallbladder

US (Figure 1) demonstrated mild gallbladder wall thickening with no gallstones, gallbladder sludge, or pericholecystic fluid. MRCP was significant for acute, possibly acalculous cholecystitis with possible concomitant duodenitis and pancreatitis and no choledocholithiasis or biliary tree dilatation.

**Figure 2:**
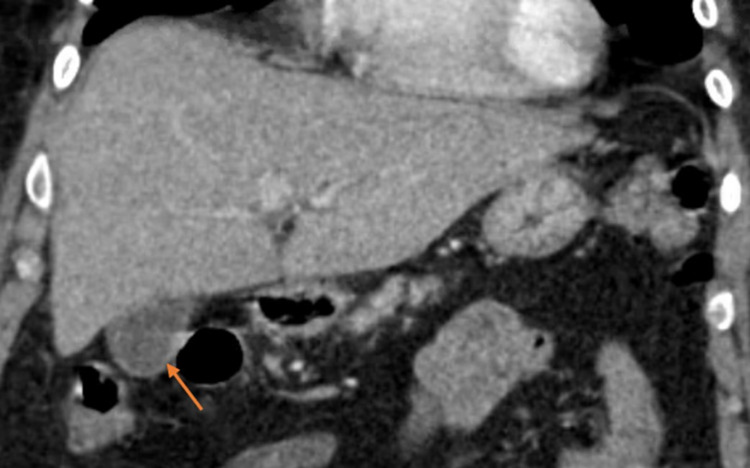
CT image demonstrating enhancing GB wall thickening (arrow) in the coronal plane with no evidence of gallstones CT - computed tomography; GB - gallbladder

**Figure 3:**
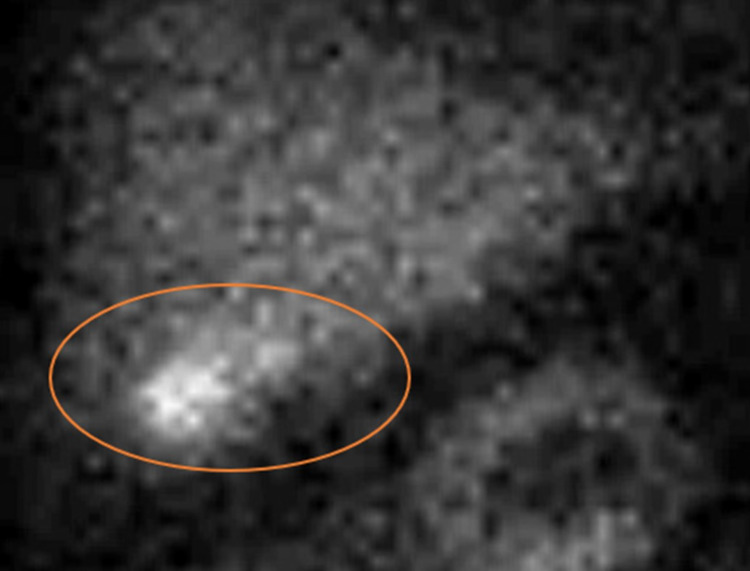
HIDA scan demonstrating radiotracer uptake into the GB (circle) with no evidence of cystic duct obstruction or gallstones HIDA - hepatobiliary iminodiacetic acid; GB - gallbladder

CT A/P (Figure 2) showed mucosal enhancement and gallbladder wall thickening with pericholecystic fluid, suggesting acute acalculous cholecystitis. HIDA scan (Figure 3) showed no cholecystitis or gallbladder wall thickening and normal gallbladder ejection fraction with no dyskinesia. 

The patient was started on empiric antibiotic therapy with clinical improvement. Upon re-assessment of his history, the patient stated he and his family recently returned from a trip to Virginia, where they hiked in an area with dense vegetation. An infectious disease workup was then performed, with the patient testing positive for *Babesia microti* (parasitemia of 1%) and Lyme IgM, but negative Lyme PCR (Table 1). Babesia was found on a peripheral blood smear, and subsequent antibody testing was positive as well. He was then treated with atovaquone 750 mg PO twice a day, azithromycin 500mg IV daily, and doxycycline 100 mg PO BID, with significant clinical improvement and eventual complete recovery from the acute febrile illness. 

## Discussion

Human babesiosis is an infectious disease caused by species of Babesia, which are zoonotic protozoa with a tropism for erythrocytes, whose primary vector is the *Ixodes scapularis* tick [1-4]. Approximately 1,000 to 2,000 individuals are diagnosed with babesiosis in the United States annually, with disease incidence rising steadily [1,4]. Over 90% of patients with babesiosis reside in the Northeastern and Midwestern United States. The white-footed mouse acts as a reservoir for the parasites, white-tailed deer act as intermediate hosts, and humans are accidental hosts [2]. The common causative agents of human babesiosis are *B. microti*, *B. divergens*, *B. duncani*, and *B. venatorum*, with *B. microti* being the most common overall [1-6]. 

Symptomatic disease results from cyclic erythrocyte invasion by merozoites, resulting in hemolysis, as well as the immune response, including proinflammatory cytokines, macrophages, and T-cells, against the pathogen and infected erythrocytes [7]. The spleen's attempts to remove infected erythrocytes result in splenomegaly. Furthermore, infected erythrocytes can adhere to vascular endothelium, resulting in tissue ischemia [7]. 

Symptoms range from asymptomatic infection to end-organ damage. Mild symptomatic infection may include waxing and waning fever, fatigue, malaise, and myalgia [7]. Anemia is common and occurs as a result of parasite-induced hemolysis, with elevated lactate, elevated reticulocyte count, and low haptoglobin. Severe disease is common in patients older than 60 years, those with hemoglobin of <10 g/dL, a history of splenectomy, concurrent Lyme disease, parasitemia >10%, parasitemia for >10 days, total bilirubin of >1.9 mg/dL or WBC count of <5.0x103/uL [3,4]. Severe disease is characterized by ARDS, renal failure, splenic rupture, heart failure, disseminated intravascular coagulation, or even acute liver failure [5,8]. Babesiosis must be suspected in patients presenting with febrile illness with recent outdoor activities in the Northeastern or Midwestern United States. If babesiosis is suspected, workup begins with a peripheral blood smear, with Wright-Giemsa staining demonstrating round or oval trophozoites, intraerythrocytic ring forms, or merozoites in Maltese cross-shaped tetrads [1,6,7,8]. Babesia PCR is more sensitive than peripheral smear and can detect the specific species [1,7]. This patient also tested positive for Lyme IgM. This is suggestive of early Lyme disease (LD); however, it is established that Lyme serology, IgG, and even IgM tests, can be positive for years after initial infection [8]. As this patient's symptomatology and laboratory findings were consistent with babesiosis and not with any known presentation of Lyme disease, it is unlikely that his current presentation was due to LD.

Treatment of babesiosis involves 7-10 days of atovaquone and azithromycin, or alternatively clindamycin and quinine [1,3,4,9]. Other regimens can be used in disease unresponsive to primary therapy, including atovaquone-proguanil; atovaquone, clindamycin or azithromycin, and doxycycline; or atovaquone, artemisinin, and doxycycline [7]. Immunocompromised patients, those with a parasitemia of 10% or greater, those with hemoglobin of <10 mg/dL or those with organ failure may require red blood cell exchange transfusion [1-4,7,9]. 

Acute acalculous cholecystitis (AAC) denotes severe gallbladder inflammation, usually due to critical illness [9]. Acute acalculous cholecystitis is uncommon in babesiosis, and its pathogenesis is poorly understood. We hypothesize that this case of AAC could have been caused by aggregation of Babesia-infected erythrocytes in gallbladder microcirculation, which would have resulted in ischemia and necrosis of the gallbladder wall [7,10]. 

## Conclusions

Babesiosis is caused by species of *Babesia*, which are zoonotic protozoa that infect red blood cells, whose primary vector is the Ixodes tick. Diagnosis should be suspect in patients presenting with a febrile illness and recent travel history to Babesia-endemic areas. Babesiosis is initially diagnosed by peripheral blood smear, followed by PCR. Treatment involves the combination of clindamycin and quinine or atovaquone and azithromycin. Acute acalculous cholecystitis is uncommon in babesiosis and may result from occlusion of gallbladder microvasculature by Babesia-infected erythrocytes. 
